# Selective Apoptotic Effect of Plasma Activated Liquids on Human Cancer Cell Lines

**DOI:** 10.3390/molecules26144254

**Published:** 2021-07-13

**Authors:** Dominika Sersenová, Zdenko Machala, Vanda Repiská, Helena Gbelcová

**Affiliations:** 1 Faculty of Mathematics, Physics and Informatics, Comenius University in Bratislava, 842 48 Bratislava, Slovakia; 2 Institute of Medical Biology, Genetics and Clinical Genetics, Faculty of Medicine, Comenius University in Bratislava, 811 08 Bratislava, Slovakia; vanda.repiska@fmed.uniba.sk (V.R.); helena.gbelcova@fmed.uniba.sk (H.G.)

**Keywords:** cold plasma, plasma-activated liquid, cancer cell, melanoma, fibroblast

## Abstract

Plasma medicine is a new field focusing on biomedical and clinical applications of cold gas plasmas, including their anticancer effects. Cold plasmas can be applied directly or indirectly as plasma-activated liquids (PAL). The effects of plasma-activated cell growth medium (PAM) and plasma-activated phosphate buffered saline (PAPBS) were tested, using a plasma pen generating streamer corona discharge in ambient air, on different cancer cell lines (melanoma A375, glioblastoma LN229 and pancreatic cancer MiaPaCa-2) and normal cells (human dermal fibroblasts HDFa). The viability reduction and apoptosis induction were detected in all cancer cells after incubation in PAL. In melanoma cells we focused on detailed insights to the apoptotic pathways. The anticancer effects depend on the plasma treatment time or PAL concentration. The first 30 min of incubation in PAL were enough to start processes leading to cell death. In fibroblasts, no apoptosis induction was observed, and only PAPBS, activated for a longer time, slightly decreased their viability. Effects of PAM and PAPBS on cancer cells showed selectivity compared to normal fibroblasts, depending on correctly chosen activation time and PAL concentration, which is very promising for potential clinical applications. This selectivity effect of PAL is conceivably induced by plasma-generated hydrogen peroxide.

## 1. Introduction

Plasma medicine is a relatively new interdisciplinary field of medicine where cold atmospheric plasmas lead to therapeutic effects in the human organism. Cold plasma has already been used in some countries to sterilize medical equipment [1] or to enhance wound healing [2]. More clinical applications are being developed including cancer therapy [3,4].

Recently, the focus has been on indirect plasma treatment with so-called plasma-activated (stimulated) liquids (PAL)—liquids that were treated with plasma and subsequently added to cells. Upon PAL application, the only factors affecting the cells are reactive oxygen and nitrogen species (RONS) [5], which are considered to be key agents in the plasma therapy [6]. RONS naturally play an important role in many physiological processes, but it is difficult to precisely isolate their function. Although a low level of RONS also contributes to cell proliferation, the effect of higher concentrations of reactive oxygen species (ROS) is already employed in various cancer therapies, for example in radiotherapy, photodynamic therapy or some types of chemotherapy [7]. The action of plasma-generated RONS in PAL is generally milder because RONS are primarily generated only in an extracellular environment. Their interaction with the cells can lead to intracellular ROS increase and induce different cellular responses, including activation of apoptotic signaling pathways. Dynamics of chemical species evolution in plasma discharge is very complex. Therefore, the exact types of RONS or their chemical reactions have to be studied in more detail [8] along with their interaction with liquids and living cells or tissues. The most important RONS generated in PAL are H_2_O_2_, ·OH, HO_2_·, O, ^1^O_2_, O_2._^−^, O_3_, ·NO, NO_2_, NO_2_^−^, NO_3_^−^, and ONOO^−^. They can couple with reactive chlorine species if the liquid environment contains chlorine ions [9,10,11,12]. Hydrogen peroxide, H_2_O_2_, alone in higher concentrations acts cytotoxically, but when a more specific and selective effect is required, other mechanisms must be applied. The synergistic effect of H_2_O_2_ and nitrite NO_2_^−^ was shown when killing cancer cells [7,9,10,11] while the role of nitrate NO_3_^−^ is not clearly established. Typically, no additional effect of NO_3_^−^ was observed [11,12], although in some studies a different cell viability was achieved by varying NO_3_^−^ concentration [13]. Short-lived peroxynitrite ONOO^−^ and singlet delta oxygen ^1^O_2_ also play important roles in contribution to anticancer effects [9,14].

In general, the right plasma treatment specific for each application, plasma source and geometry should be identified [3,13]. In most studies with cancer cells, plasma is generated by plasma jets using noble gases (He, Ar). RONS are then produced as the plasma effluent emerges into the surrounding air and their composition is usually different than in discharges generated directly in ambient air.

PAL have potential in clinical anticancer applications and are safer for a patient than a direct plasma treatment as no direct contact with or proximity to high voltage is needed. PAL can be injected into the tissues, even where the direct plasma treatment is not possible, for example in peritoneal or micrometastases treatment. PAL keep their anticancer properties for a longer time, so they can be prepared in advance and stored [15,16].

The studies on plasma-activated cell growth medium (PAM) conducted in the last decade [17] showed that PAM can reduce cell viability in various cancer cell lines—glioblastoma [17,18,19], breast cancer [16,17,18,19], bladder cancer [20], lung cancer [16], hepatic cancer [16], and colorectal [21] or ovarian cancer [22] cells, including chemoresistant ovarian carcinoma cells [23]. Plasma application does not seem to cause as many side effects as the current cancer therapies and the in vitro and first in vivo animal experiments propose its universal use on different cancer types. Recent studies revealed that plasma can induce immunogenetic cell death by expressing damage-associated molecular patterns (for example calreticulin) in the cancer cells after plasma treatment [24]. This means that the clinical application of plasma (and PAL) might lead to better results than those observed in vitro or in vivo on immunosuppressed mice, because the patient’s own immune system could contribute to fighting against the cancer cells.

Although the anticancer effects of PAM are very promising, detailed insight into their biochemical activity are difficult because of their complex composition and many possible reactions between RONS and medium compounds. Therefore, other, simpler liquids, such as phosphate buffer saline (PBS), are being studied, although there are still not as many studies on plasma-activated PBS (PAPBS) as on PAM [12,25]. Another advantage of PBS in in vitro experiments is that it is not cell line specific, as the cell growth media, and if the plasma treatment is not excessive, the pH of this buffered solution remains physiological (plasma-activated water or other non-buffered solutions typically become acidic after plasma treatment [14]). PAPBS was shown capable of decreasing viability in glioblastoma cells [12,25] or pancreatic cancer cells [25].

In this work we focused on the effects of PAM and PAPBS. Both PAM and PAPBS were activated by a portable plasma pen operating with streamer corona discharge in ambient air, unlike most similar studies that typically use plasma jets operating with noble gases. Ambient air used as a plasma generating gas is cheaper, broadly available, and is a good precursor of RONS. Furthermore, in our conditions it leads to approximately 1:1 ratio of H_2_O_2_ and NO_2_^−^ generated in PAPBS, which is presumably an optimum stoichiometric ratio for their mutual reaction leading through peroxynitrite to further RONS [9,26,27]. This aspect, together with operating in just ambient air, distinguishes our plasma pen from other plasma sources and makes it potentially easily applicable in future clinical practise worldwide. The same plasma source and PAM have been already successfully tested in vitro to induce apoptosis in several cancer cell lines and the key possible mechanisms responsible for the plasma and PAM selectivity have been identified, especially the role of secondary ^1^O_2_ emerging from the mutual chemistry of H_2_O_2_ and NO_2_^−^ in PAM, and inactivating the protective catalase on the cancer cell membranes [9,28,29]. 

Our focus is set primarily on the effect of plasma-activated liquids on human melanoma cells A375. Melanoma is one of the most aggressive types of cancer and the deadliest skin cancer with around 200,000 new cases detected annually [30]. The occurrence of melanoma on human skin makes it an easy target for plasma treatment with no surgical requirements. Even after metastases spread, PAL injections could be potentially applicable. Besides the cell viability change upon exposure to PAM and PAPBS, the apoptosis induction was also investigated. Apoptosis, or programmed cell death, is an important and active regulatory pathway of cell growth and proliferation in healthy tissues. After plasma treatment, it is a desirable cell death type of cancer cells, because it does not cause significant inflammatory response in an organism [31]. In most studies that investigated plasma- or PAL-induced cell death type, apoptosis was confirmed [32], although necrosis may also occur [33]. 

Another still unclarified question is the exact mechanisms of cell signaling pathways, which are activated by plasma to induce cell death. Two main points in intracellular signaling are caspases and mitochondria, therefore the activity of caspase 3 and 7, and a change in mitochondrial membrane potential, were studied here. Caspases are cysteine proteases with important roles in apoptosis propagating process in response to proapoptotic signals. The effector caspases, such as caspase 3 and 7, act further downstream and direct cellular breakdown through a cleavage of structural proteins. Activation of these caspases is thus a hallmark of apoptosis. Mitochondria are crucial cell organelles and contain key regulators of cell-death processes. Mitochondrial membrane potential changes have been implicated in apoptosis, necrotic cell death, and caspase-independent cell-death processes. Depolarization of the inner mitochondrial membrane potential is thus an indicator of mitochondrial dysfunction and cellular health, which has become increasingly important in the study of apoptosis, drug toxicity and multiple disease states. 

PAL in this article were also applied on normal dermal fibroblasts to investigate the potential selective effect of PAL treatment against cancer cells. Two other cancer cell lines (pancreatic cancer and glioblastoma cells) were also examined to assess the potential effects of air plasma-generated PAL in cancer therapy in a more general manner.

## 2. Results

### 2.1. RONS Concentration in PAPBS

**Figure 1:**
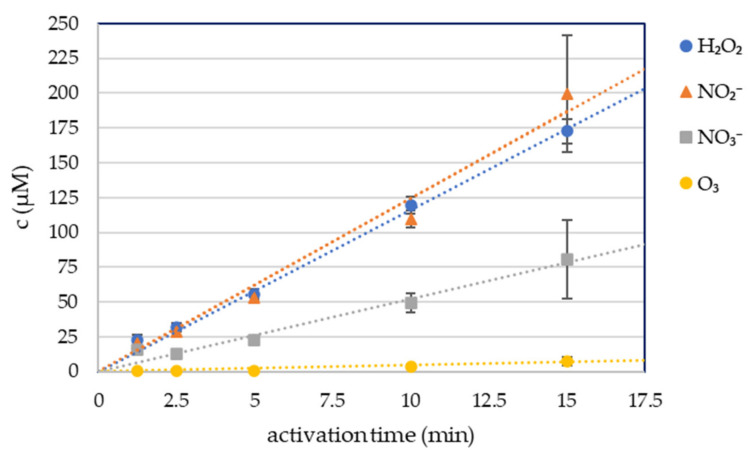
Hydrogen peroxide, nitrite, nitrate and ozone concentration in plasma-activated PBS (PAPBS).

Four long-lived RONS in plasma-activated phosphate buffered solution were measured: hydrogen peroxide H_2_O_2_, nitrites NO_2_^−^, nitrates NO_3_^−^ and dissolved ozone O_3_, with spectrophotometric colorimetric methods (Figure 1). 5 mL of PBS was treated for 1.25, 2.5, 5, 10, and 15 min with the streamer corona discharge in ambient air under the same conditions as the cell growth media and RONS were measured immediately after the plasma activation. The streamer corona discharge in ambient air was applied at low power (P ≈ 0.4 W), therefore even 15 min treatment of the liquid did not increase its temperature. The buffering capacity of PBS also kept the pH unchanged after the treatment.

The concentrations of the plasma-produced H_2_O_2_ and NO_2_^−^ were approximately equal at each condition. The NO_3_^−^ concentration was significantly lower. The concentration of these RONS increased linearly with the time of plasma activation. 

The O_3_ concentration was negligible even after 15 min of plasma activation.

### 2.2. PAL Effects on the Cell Viability

We evaluated the indirect effect of plasma-activated liquids on the cells by measuring the cell viability. Viability was measured 24 h from adding PAL. Analysis at 6, 48 or 72 h after PAL application was performed too. However, after 6 h the effect just started to be observable and time periods longer than 24 h of cell incubation were too long for apoptosis tests, as after this time most of the cells were in the late apoptosis state (as presented in Section 2.3) and started to disintegrate. Therefore, all presented data are from the viability analysis 24 h after PAL application. To compare the treatment with 100% PAPBS or 50% PAPBS, we added secondary controls incubated in untreated PBS in same concentration. 

**Figure 2:**
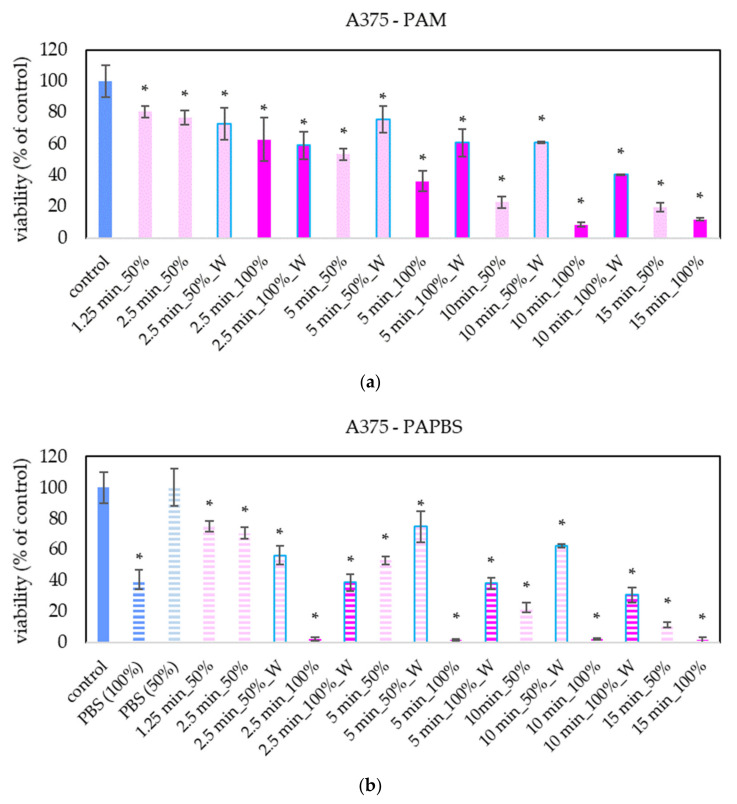
Effect of PAL on cell viability of human melanoma cells A375 24 h after PAL application. Plasma-activated medium (PAM) (**a**) or plasma-activated PBS (PAPBS) (**b**) activated for 1.25, 2.5, 5, 10 or 15 min at 100% or 50% (1:1 dilution) concentration were used. Time of PAL action was 24 h (without blue frames) or 30 min, after which PAL were replaced with untreated medium (“washing”, W, blue frames). The significant results (*p* < 0.05) are marked with *. 100% PAPBS, resp. 50% PAPBS was tested compared to in 100% PBS, resp. 50% PBS, significance of other samples compared to control. Viability was normalized to the control cells incubated in the medium.

The focus of our work was on melanoma A375 cells. The “dose” effect of the PAL can be clearly seen—with the increasing PAL concentration and with the increasing activation time, the decrease of cell viability was more significant (Figure 2). We observed this trend even in 25% and 12.5% PAL dilution (these data are not presented in the graphs to keep them readable). In most samples, the effects of PAM and PAPBS on cell viability are comparable. The effects of 100% PAPBS seems to be most effective and decreased the cell viability almost to 0% in all samples with no differences between activation times. This effect is partly caused by reducing viability because of cultivation in long PBS, but the viability decrease is still significant. Furthermore, only 30 min of incubation in PAM or PAPBS was enough to start processes leading to the decreased viability in cells. 

**Figure 3:**
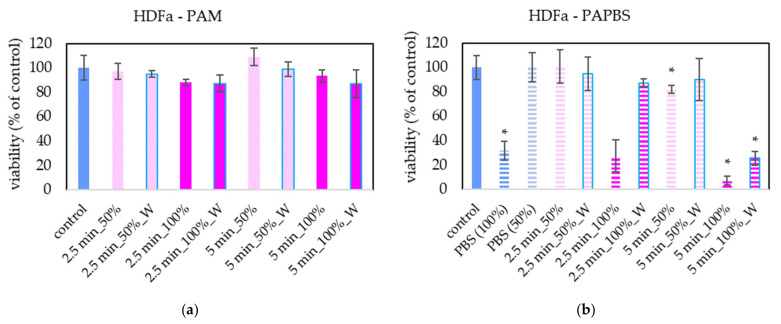
Effect of PAL on cell viability of human dermal fibroblasts HDFa 24 h after PAL application. Plasma-activated medium (PAM) (**a**) or plasma-activated PBS (PAPBS) (**b**) activated for 2.5 or 5 min in 100% or 50% concentration were used. Time of PAL action was 24 h (without blue frames) or 30 min, after which PAL were replaced with untreated medium (“washing”, W, blue frames). The significant results (*p* < 0.05) are marked with *. Significance of 100% PAPBS, resp. 50% PAPBS was tested compared to in 100% PBS, resp. 50% PBS, significance of other samples compared to control. Viability was normalized to the control cells incubated in the medium.

PAM and PAPBS were applied on healthy skin cells (human dermal fibroblasts HDFa) to compare with their effects on melanoma cells (Figure 3). PAL activated for 2.5 min did not significantly affect the cell viability of HDFa fibroblasts. We did not observe a significant viability change either after application of PAM activated for a longer time. Only PAPBS activated for 5 min led to their viability decrease when it was incubated with cells for 24 h. In the case of 100% PAPBS, the 30 min incubation was enough to induce the viability change. On the other hand, in some samples, we also detected the opposite effect, i.e., a slight cell viability enhancement (mostly in 50% of 5 min treated PAM), but this effect was not statistically significant.

**Figure 4:**
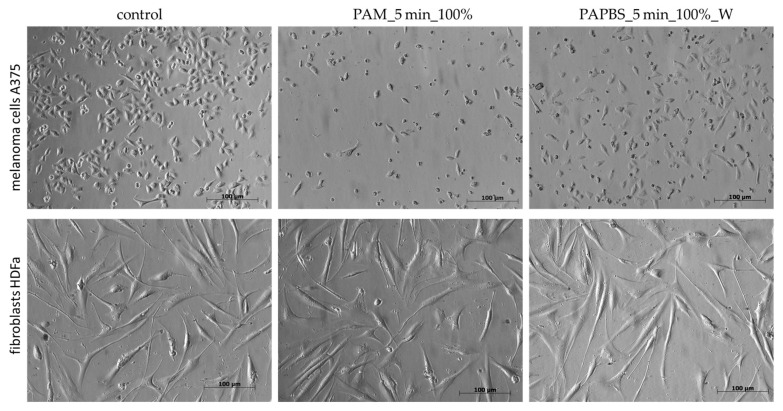
Effect of PAL on morphology of melanoma cells A375 and dermal fibroblasts HDFa, 24 h after PAL application. PAL were activated for 5 min. Two PAL treatments are showed: 100% plasma-activated medium (PAM) or 100% plasma-activated PBS (PAPBS) incubated with cells for 30 min and then replaced with the untreated medium (“washing”, W). (Zeiss Axio Verte.A1 optical microscope, Zeiss, Jena, Germany).

The morphological change of the cells after 24 h of incubation with PAL was also notable (Figure 4). In PAL samples, a significant amount of the cells was rounded, detached from the bottom, and a nuclear condensation was observed. On the other hand, no morphological changes were noted on HDFa cells.

**Figure 5:**
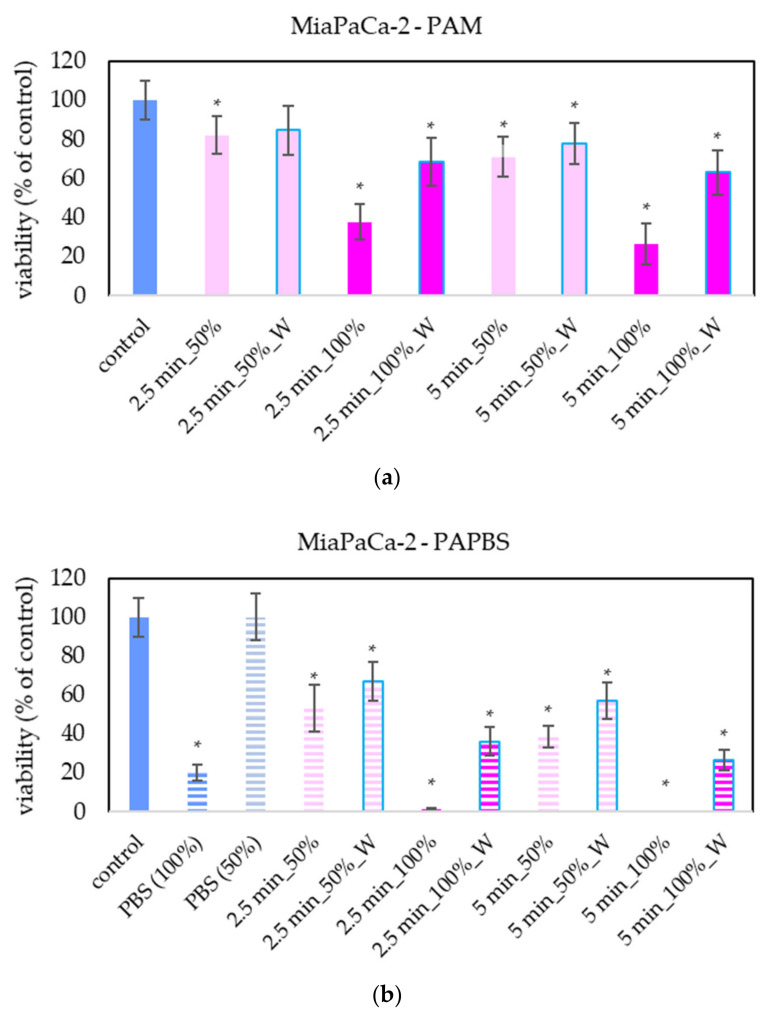
Effect of PAL on cell viability of pancreatic cancer cells 24 h after PAL application. Plasma-activated medium (PAM) (**a**) or plasma-activated PBS (PAPBS) (**b**) activated for 2.5 or 5 min in 100% or 50% concentration were used. Time of PAL action was 24 h (without blue frames) or 30 min, after which PAL were replaced with untreated medium (“washing”, W, blue frames). The significant results (*p* < 0.05) are marked with *. Significance of 100% PAPBS, resp. 50% PAPBS was tested compared to in 100% PBS, resp. 50% PBS, significance of other samples compared to control. Viability was normalized to the control cells incubated in the medium.

The effect of PAL on A375 and HDFa was compared with other cancer cell lines: pancreatic cancer cells MiaPaCa-2 (Figure 5). The advantage of MiaPaCa-2 cells is that they are cultivated in the same medium as melanoma A375 cells, so the possible difference in the effect would be only due to different properties of the cells and not due to the different medium composition. In terms of viability, the effect of PAL on MiaPaCa-2 cells was mostly comparable with the effect on A375 cells.

**Figure 6:**
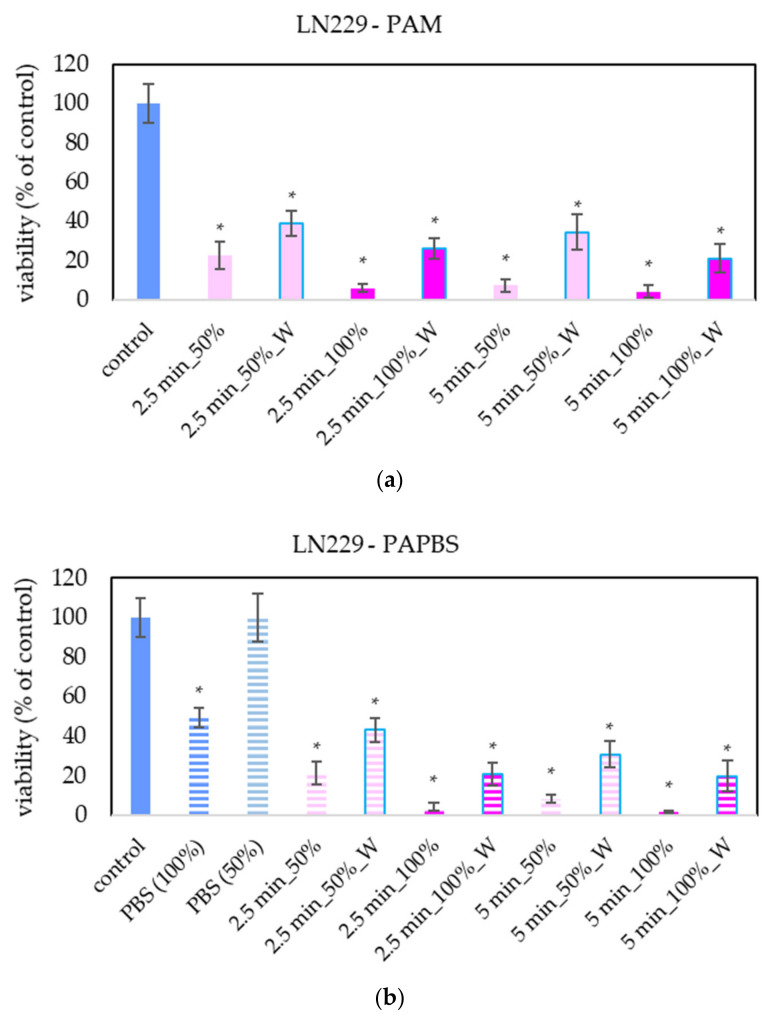
Effect of PAL on cell viability of glioblastoma cells LN229 24 h after PAL application. Plasma-activated medium (PAM) (**a**) or plasma-activated PBS (PAPBS) (**b**) activated for 2.5 and 5 in 100% or 50% concentration were used. Time of PAL action was 24 h (without blue frames) or 30 min, after which PAL were replaced with untreated medium (W, blue frames). The significant results (*p* < 0.05) are marked with *. Significance of 100% PAPBS, resp. 50% PAPBS was tested compared to in 100% PBS, resp. 50% PBS, significance of other samples compared to control. Viability was normalized to the control cells incubated in the medium.

For LN229 cell cultivation, a slightly different brand of medium was used, although it was still the same type of high-glucose DMEM. LN229 were the most sensitive cells to the antiproliferative effect of PAL, in which even the PAL treated for 2.5 min reduced the cell viability to 20% or less (Figure 6).

### 2.3. PAL Effects on the Apoptosis Induction

To further analyse the effect of plasma-activated liquids on the induction of cell apoptosis we used annexin V and 7-AAD staining. By combination of these dyes, it is possible to distinguish live, early apoptotic, late apoptotic (dead) cells and non-apoptotic dead cells. 

We focused mostly on plasma treatment and analysis of melanoma A375 cells; therefore, more different PAL treatments on these cells were performed. In the case of other cells, only the most promising treatments, based on the viability results, were chosen. 

**Figure 7:**
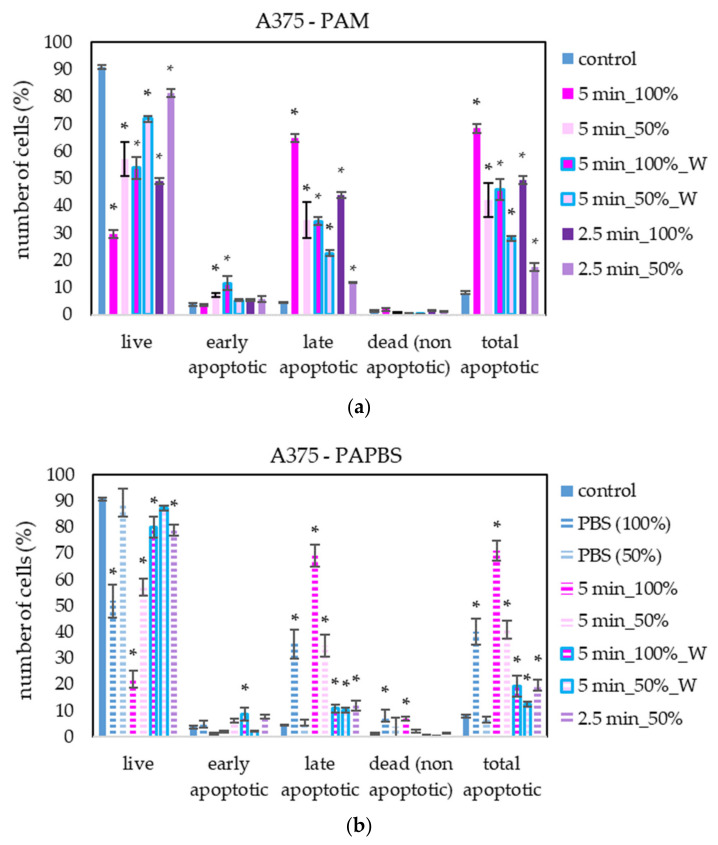
The effect of PAL on apoptosis induction in human melanoma cells A375, measured 24 h after PAL application. Plasma-activated medium—PAM (**a**) or plasma-activated PBS—PAPBS (**b**) activated for 2.5 or 5 min in 100% or 50% concentration were used. Time of PAL action was 24 h (without blue frames) or 30 min, after which PAL were replaced with untreated medium (“washing”, W, blue frames). The significant results (*p* < 0.05) are marked with *. Significance of 100% PAPBS, resp. 50% PAPBS was tested compared to in 100% PBS, resp. 50% PBS, significance of other samples in PAM was tested compared to control.

PAM and PAPBS activated for 2.5 and 5 min induced apoptosis in A375 cells (Figure 7), both in 100% and 50% concentration and even when PAL were replaced with the untreated medium after 30 min (W). The “dose” effect is observable. Almost all apoptotic cells were already considered dead 24 h after PAL application. A very low amount of the cells in non-apoptotic death (around 8%) was observed when 100% PBS or PAPBS for 24 h was applied, whereas the effects of PAPBS and PBS were comparable. This indicated that the effect in this case is most likely caused by non-physiological conditions of the pure PBS, because there is no additional increase in dead non-apoptotic cells due to the plasma treatment in PAPBS. 

**Figure 8:**
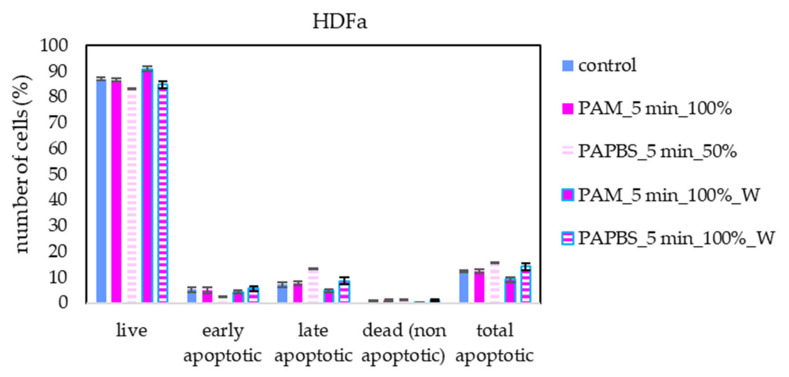
The effect of PAL on apoptosis induction in human dermal fibroblasts HDFa, measured 24 h after PAL application. Plasma-activated medium (PAM) or plasma-activated PBS (PAPBS) activated for 5 min in 100% or 50% concentration were used. Time of PAL action was 24 h (without blue frames) or 30 min, after which PAL were replaced with untreated medium (“washing”, W, blue frames). No significant change was observed.

Apoptosis or necrosis in normal fibroblasts HDFa were not detected in our experiments (Figure 8). Here, we analysed 100% PAPBS only when it was applied for 30 min, then replaced with untreated medium (W) since these primary cells were not capable of surviving in the 100% PBS for 24 h (even untreated by plasma). Although in viability experiments, the cell viability after applying PAPBS treated for 5 min (50% or 100%_W) decreased, stronger PAPBS could suppress proliferation of HDFa cells without leading to cell death.

**Figure 9:**
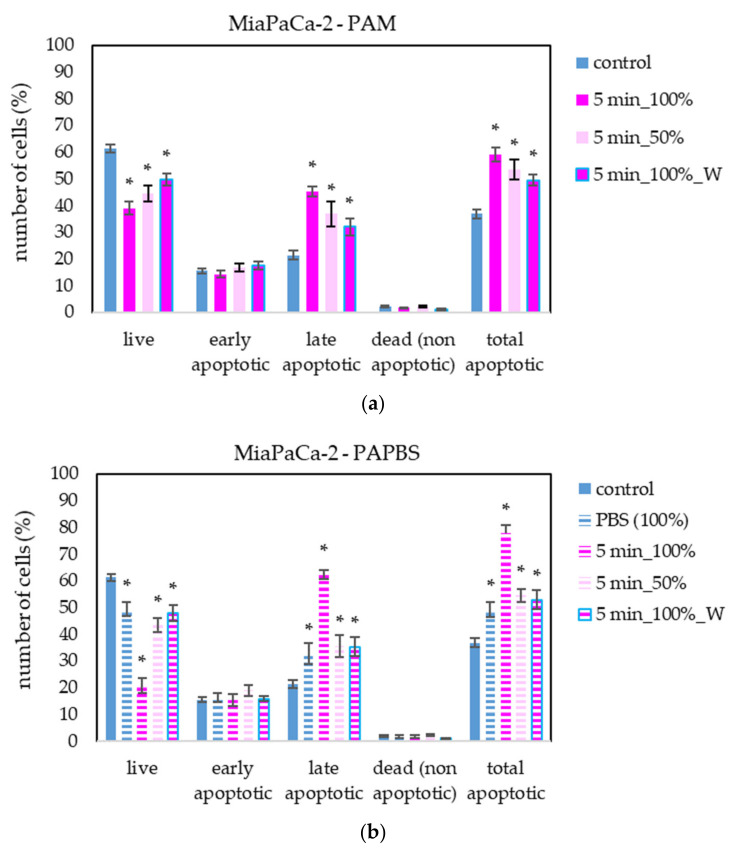
The effect of PAL on apoptosis induction in pancreatic cancer cells MiaPaCa-2, measured 24 h after PAL application. Plasma-activated medium—PAM (**a**) or plasma-activated PBS—PAPBS (**b**) activated for 5 min in 100% or 50% concentration were used. Time of PAL action was 24 h (without blue frames) or 30 min, after which PAL were replaced with untreated medium (“washing”, W, blue frames). The significant results (*p* < 0.05) are marked with *. Significance of 100% PAPBS was tested compared to cells in 100% PBS, significance of other samples was tested compared to control.

**Figure 10:**
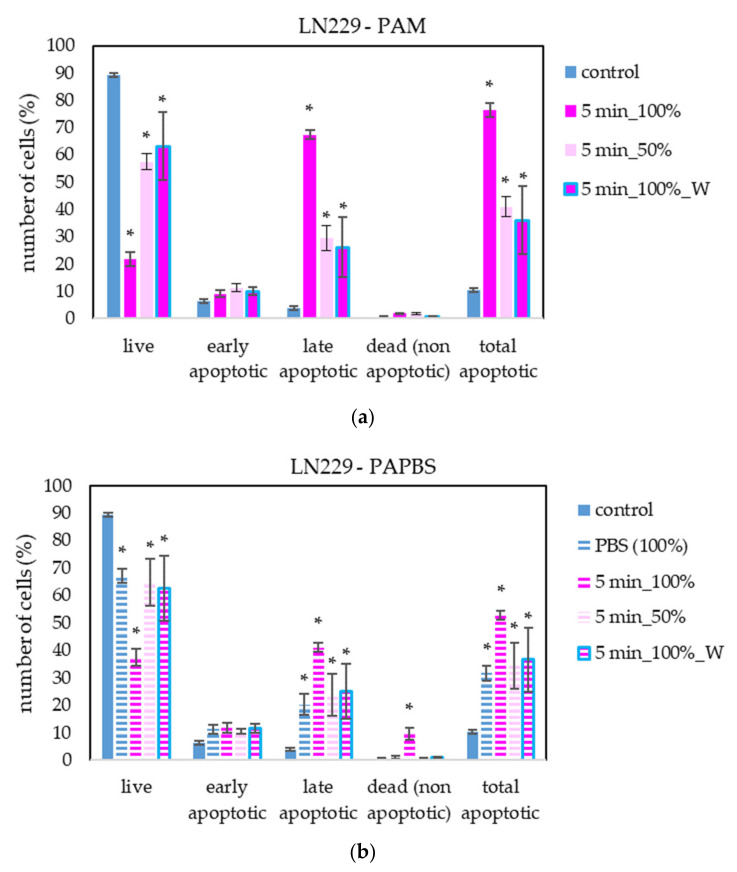
The effect of PAL on apoptosis induction in glioblastoma cells LN229, measured 24 h after PAL application. Plasma-activated medium—PAM (**a**) or plasma-activated PBS—PAPBS (**b**) activated for 5 min in 100% or 50% concentration were used. Time of PAL action was 24 h (without blue frames) or 30 min, after which PAL were replaced with untreated medium (“washing”, W, blue frames). The significant results (*p* < 0.05) are marked with *. Significance of 100% PAPBS was tested compared to cells in 100% PBS, significance of other samples was tested compared to control.

The apoptosis induction in MiaPaCa-2 and LN229 cells after PAM and PAPBS treatment was tested, too (Figure 9 and Figure 10). Although in MiaPaCa-2, there was a high percentage of apoptotic cells, even in control samples, enhancement in apoptotic cells after PAL application was significant.

PAL also successfully induced apoptosis in LN229 cells, but according to the debris amount in the sample, many cells were already disintegrated; therefore, the percentage of apoptotic cells in the time of analysis was lower than in the other cancer cell lines. LN229 cells seem to be very sensitive to PAL and we observed around 10% probably necrotic cells after incubation in 100% PAPBS activated for 5 min.

### 2.4. The Effect of PAL on Caspase 3 and Caspase 7 Activity

**Figure 11:**
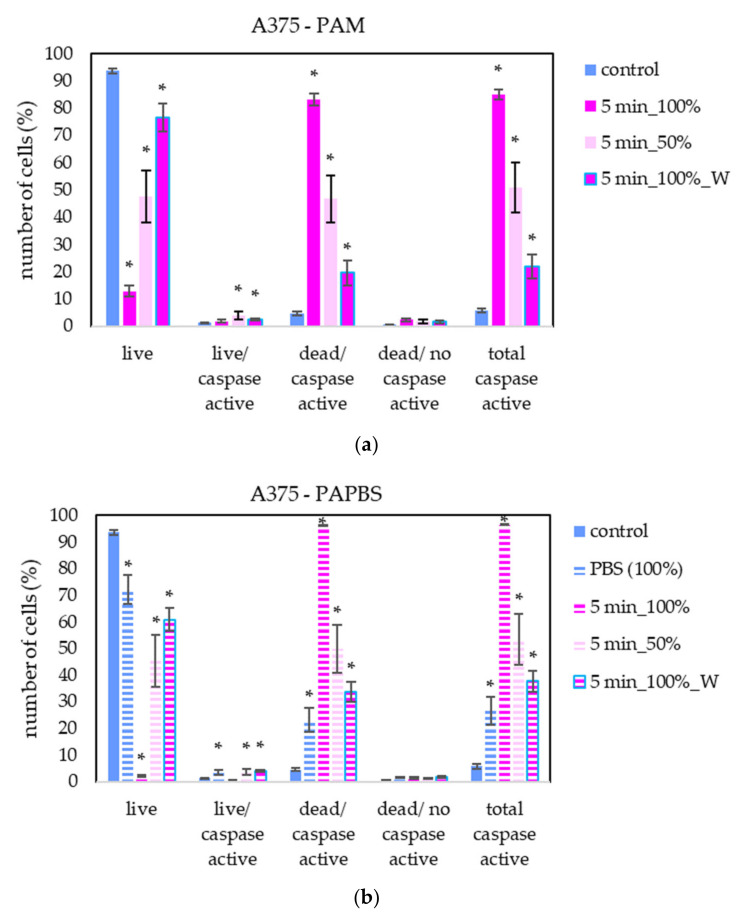
The effect of PAL on caspases 3/7 activity in human melanoma cells A375, measured 24 h after PAL application. Plasma-activated medium—PAM (**a**) or plasma-activated PBS—PAPBS (**b**) activated for 5 min in 100% or 50% concentration were used. Time of PAL action was 24 h or 30 min, after which PAL were replaced with untreated medium (“washing”, W, blue frames). The significant results (*p* < 0.05) are marked with *. Significance of 100% PAPBS was tested compared to cells in 100% PBS, significance of the other samples was tested compared to control.

To better understand the apoptotic induction and its pathway activated in cancer cells after application of PAL, the activity of caspases 3 and 7 in melanoma A375 cells was studied (Figure 11). Here, only the most promising PAL treatments, based on previously shown results, were chosen—PAM and PAPBS were activated for 5 min and applied in 100% or 50% concentration.

A significant number of cells with caspase activity, mostly already dead, were detected. In agreement with apoptosis analysis by annexin V, a significant increase in cells in early apoptosis state was detected when the diluted PAL was applied or the incubation with PAL was only 30 min.

### 2.5. The Effect of PAL on Mitochondrial Membrane Depolarisation

**Figure 12:**
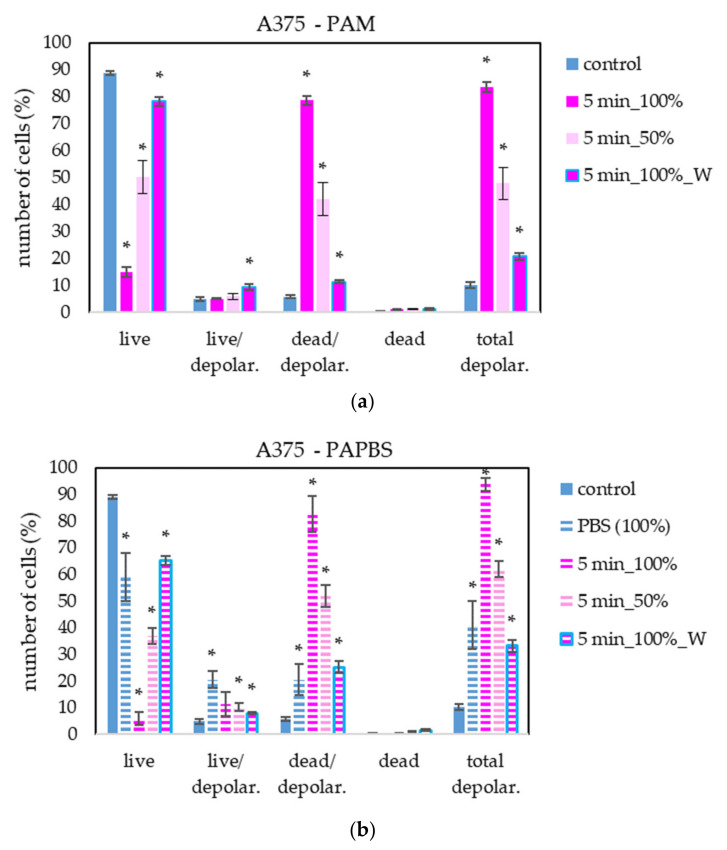
The effect of PAL on mitochondrial membrane depolarisation in human melanoma cells A375, measured 24 h after PAL application. Plasma-activated medium—PAM (**a**) or plasma-activated PBS—PAPBS (**b**) activated for 5 min in 100% or 50% concentration were used. Time of PAL action was 24 h (without blue frames) or 30 min, after which PAL were replaced with untreated medium (“washing”, W, blue frames). The significant results (*p* < 0.05) are marked with *. Significance of 100% PAPBS was tested compared to cells in 100% PBS, significance of the other samples was tested compared to control.

To provide more information about PAL’s effect on cancer with a focus on the cell death pathway, we measured the loss of mitochondrial membrane potential (depolarisation). This event is often coincident with apoptosis induction but can also occur in necrosis or caspase-independent cell death pathways. We focused on mitochondrial membrane potential change in melanoma A375 cells after treating with PAL activated for 5 s (Figure 12). The results are in good agreement with the previous apoptosis test. The most significant difference is in the higher percentage of live cells with depolarised mitochondrial membrane after PAPBS application.

### 2.6. The Effect of H_2_O_2_ and NO_2_^−^ on the Cells

**Figure 13:**
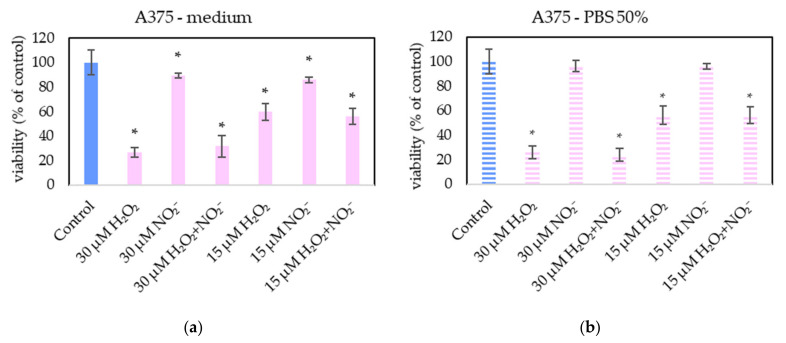
The effect of H_2_O_2_ and NO_2_^−^ on A375 cells viability after 24 h incubation. H_2_O_2_ and NO_2_^−^ were externally added into 100% medium (**a**) or 50% PBS diluted with medium (**b**). Significance of was tested compared to cells in 100% medium or 50% PBS, no significant difference between only H_2_O_2_ and H_2_O_2_ + NO_2_^−^ was found. Viability was normalized to the control cells incubated in the medium. The significant results (*p* < 0.05) are marked with *.

**Figure 14:**
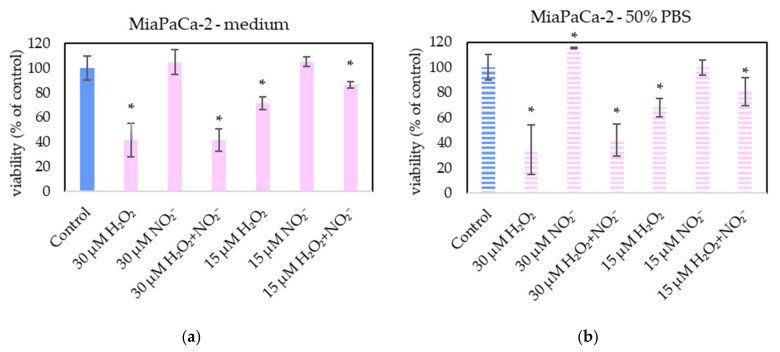
The effect of H_2_O_2_ and NO_2_^−^ on MiaPaCa-2 cells viability after 24 h incubation. H_2_O_2_ and NO_2_^−^ were externally added into 100% medium (**a**) or 50% PBS diluted with medium (**b**). Significance of was tested compared to cells in 100% medium or 50% PBS, no significant difference between only H_2_O_2_ and H_2_O_2_ + NO_2_^−^ was found. Viability was normalized to the control cells incubated in the medium. The significant results (*p* < 0.05) are marked with *.

**Figure 15:**
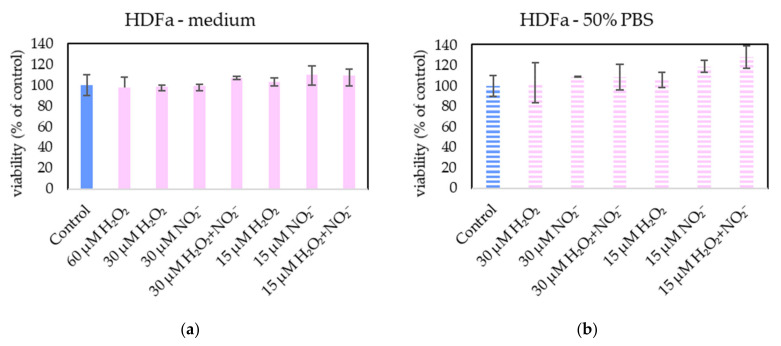
The effect of H_2_O_2_ and NO_2_^−^ on HDFa cells viability after 24 h incubation. H_2_O_2_ and NO_2_^−^ were externally added into 100% medium (**a**) or 50% PBS diluted with medium (**b**). No significant change was found. Viability was normalized to the control cells incubated in the medium.

In order to examine the roles of H_2_O_2_ and NO_2_^−^ in PAL, external H_2_O_2_ and NO_2_^−^ and their combination were added in PBS and cell medium and applied on melanoma A375 cells (Figure 13), pancreatic cancer cells MiaPaCa-2 (Figure 14) and human dermal fibroblasts HDFa (Figure 15). The comparable concentrations of H_2_O_2_ and NO_2_^−^ as measured in PAPBS were used to compare with PAPBS effect (30 µM and 15 µM). The cell viability was measured after an additional 24 h of incubation. There was no difference between the viability of cells incubated in 50% PBS (PBS diluted with medium) and cells only in medium.

The addition of H_2_O_2_ significantly decreased the viability of both cancer cell types, even in 15 µM concentration (which is comparable with PAPBS, activated for 2.5 min in 50% concentration). NO_2_^−^ in 100% medium slightly decreased the viability of melanoma cells, while an opposite effect was observed in 30 µM NO_2_^−^ in 50% PBS when applied on MiaPaCa-2.

Neither of H_2_O_2_ and NO_2_^−^ in these concentrations showed any effect on HDFa cells, even when 60 µM H_2_O_2_ in medium was applied.

## 3. Discussion

The main objective of this study was testing the effects of liquids activated by cold air plasma of streamer corona discharge: plasma-activated medium and PBS, on human melanoma cells A375 and other cancer cell lines (glioblastoma cells LN229, pancreatic carcinoma cells MiaPaCa-2) and normal non-cancerous fibroblasts (HDFa).

In addition to the biological effects induced in the studied cells, we also focused on the electrical characteristics of the plasma pen and the chemical properties of PAPBS. Although we used a high voltage (12.8 kV), the maximum current was limited to only about 25 mA, thanks to which this discharge can be painlessly applied to the skin.

We measured around 55 µM of both plasma-generated H_2_O_2_ and NO_2_^−^ in PAPBS after 5 min treatment of 5 mL in 1 cm gap and their concentrations increased approximately linearly with plasma treatment time. In the study with kINPen plasma jet operating in argon, the same treatment time and gap lead to the generation of 800 µM H_2_O_2_ and 300 µM NO_2_^−^ in 2 mL PAPBS [12]. In 500 µL PAPBS activated with helium plasma jet for 4 min, 1400 µM H_2_O_2_ and 1600 µM NO_2_^−^ were detected [11]. PAPBS activated with helium jet for 2 min per 100 µL from 2 cm distance generated 700 µM H_2_O_2_ and 350 µM NO_2_^−^ [34]. A significantly higher concentration of H_2_O_2_ than NO_2_^−^ was also measured in PAM after activating 100 µL DMEM by helium plasma jet for 1 min from 2 cm distance (500 µM H_2_O_2_ and 300 µM NO_2_^−^) [35]. 1 mL RPMI medium activated with helium jet for 1 min (U = 12 kV) contained approximately 80 H_2_O_2_ and 60 µM NO_2_^−^ [36]. On the other hand, argon plasma jet with ultra-high density of electrons generated around 17 µM H_2_O_2_ and 640 µM NO_2_^−^ in 3 mL DMEM medium after 1 min [10]. Some more plasma-generated RONS in liquids are summed up in [10]. However, comprehensive comparing of these concentrations is one of the challenges in plasma medicine research. The geometry of discharge setup strongly affects the RONS production, so the generated RONS concentrations are not only the function of the plasma source and time, but also of the liquid volume, distance between the plasma source and liquid, depth of the bowl/well, gas type and flow rate and other parameters.

Although ozone can play a role in direct plasma treatment, it was detected in very low concentrations in PAPBS activated by this streamer corona pen because of its poor solubility in liquid in this type of geometry. Different geometry could increase ozone solubility (closed reactor or electrospraying [37]); however, ozone would be quickly removed by its reaction with NO_2_^−^ [38]. More detailed physical processes and chemical reactions leading to the formation of these RONS in the streamer corona discharge in ambient air were described in our previous work [37].

Our results show that both PAM and PAPBS decreased the cell viability in all studied cancer cell lines. Since we focused mostly on melanoma cells, we studied a larger interval of plasma activation times in these cells (from 1.25 to 15 min per 5 mL). Even the shortest activation time significantly lowered the cancer cell viability, and we observed the “dose” dependency. With the increasing time and PAL concentration, the cell viability was progressively more reduced, and the 15 min treatment time reduced the viability to almost 0%, when the cells were incubated in 100% PAL. A lower but still significant decrease was observed after incubation in 50% PAL (i.e., PAL added to the growth medium in a ratio 1:1). This approach better simulates physiological conditions, when it is not possible to incubate cells only in plasma-activated liquids in real tissues, because of the present interstitial liquids.

It has been previously shown that the composition of the growth media can affect the result on the cell viability [16]. We used two cell lines that required the identical cell growth medium DMEM (A375, MiaPaCa-2), one cell line with a different brand of a very similar DMEM medium and glutamine addition (LN229), and healthy primary fibroblasts that require a different medium. The viability decreases of melanoma and pancreatic cells were comparable. The viability of LN229 cells was lowered more significantly, and we assume that it is rather due to their higher sensitivity to PAM than due to the slightly different composition of their medium.

The cell incubation in 100% PAPBS could lead to the conditions comparable with PAM, but 24 h incubation in (non-treated) PBS is not optimal for the cell growth and their viability was decreased because of the lack of nutrition. Some of the cells were able to survive 24 h incubation in pure PBS; however, all cells died in 100% PAPBS (including fibroblasts). Therefore, we performed experiments when the cells were incubated in 100% PAPBS for 30 min only and then returned into the untreated medium. With this approach, 30-min incubation in either PAM or PAPBS was enough to start cell processes leading to their lower viability and apoptosis. The first few incubation hours are considered as key for anticancer treatments [25]; however, a much shorter time is probably needed, as we also showed in our precedent work [28].

No viability reduction of non-cancerous primary fibroblasts after PAM application was observed. This indicates a certain level of the desired selectivity of this type of PAL application to cancer cells, although in some studies, viability decrease was also observed in healthy cells. Different types of human fibroblasts were used in those studies, and it was shown that the treatment with PAM was better tolerated than the direct plasma treatment [39]. PAM activated by helium plasma jet decreased the Nuff fibroblasts’ viability by 20% [40] and 3T3 fibroblasts if PAM was activated for more than 30 s [36]. PAM activated by argon plasma reduced the viability of WI-38 fibroblasts by 40% [19]; however, it had no effect on mammary epithelial cells [10] or human astrocytes [17]. This difference may be due to the different sensitivity of cells to H_2_O_2_ [41].

The viability of HDFa fibroblasts in our results slightly decreased when they were incubated in PBS activated for 5 min for all types of application—100% and 50% of PAPBS or in 100% PAPBS for 30 min only—but we did not observe any morphological changes or apoptosis induction, except in 100% PAPBS. In the study with 1 h incubation of fibroblasts in PAPBS, the viability of fibroblasts was decreased more significantly than the viability of melanoma cells [11], and therefore a much shorter time of incubation when using PAPBS is needed.

In PAM in general, the generated RONS react with medium components which in turn reduces the number of RONS interacting directly with cells, probably including the H_2_O_2_ concentration [18]. Therefore, it is possible that PAPBS affects healthy cells that are more sensitive to higher H_2_O_2_ concentrations. It was shown that normal cells are more sensitive to the induction of apoptosis with H_2_O_2_ because they are not protected with membrane-associated catalase as cancer cells [42]. On the other hand, some studies observed a stronger effect on cancer cells when PAM was added to the cells compared to PAPBS addition [25]. In this work, 50% PAL in the well induced comparable results in cancer cell lines for both PAM and PAPBS, while fibroblasts were slightly more sensitive to PAPBS and less sensitive to PAM.

Another point to discuss is different media composition. Because of their complexity, it is difficult to determine the compounds which could be responsible for increasing or decreasing cytotoxicity. FBS was shown to decrease PAM anti-cancer properties [43]; however, in our work the serum-free plasma-activated fibroblasts medium did not kill fibroblasts while other media with 10% FBS killed cancer cells. Our attempt to switch media was unsuccessful because the cells were not in good condition when cultivated in the foreign media. Since PAM did not damage fibroblasts, we assume that the mechanism of PAM cytotoxicity in case of cancer cells is not by destroying important medium cell nutrition compounds. The different RONS generation was shown comparing medium with or without serum—although H_2_O_2_ concentration was the same, significantly less NO_2_^−^ was produced in serum-free DMEM [35]. Moreover, it was shown that after more than 18 h from plasma activation, PAM lost its anti-cancer capacity and the cells proliferated again when incubated in it; therefore, the important nutrients must have been preserved [20]. However, it is possible that longer or more intensive plasma activation of media can degrade amino acids, as was shown in the work of Chauvin et al. [35].

Annexin V and 7-AAD staining confirmed the apoptosis induction in all studied cancer cell lines after all types of PAL treatments and almost no cells died in non-apoptotic pathway. On the contrary, no apoptosis in fibroblasts was detected. Furthermore, we studied the caspase 3/7 activity and mitochondrial membrane epolarization in A375 cells after PAL treatment. A significantly increased caspase 3/7 activity was detected after PAM or PAPBS application. An increased expression of caspase 3 and 8 after PAM and direct plasma treatment of HeLa cells was also observed in [39] and an expression of caspases 3, 7 and 9 after PAM treatment was shown in ovarian cancer cells [22]. Low H_2_O_2_ concentration leads to the caspase-dependent apoptosis and mitochondrial membrane potential change [44]. In a different study on gastric cancer cells, the inhibition of caspase 3 lead to stopping apoptosis induction after the plasma treatment [28]. We detected the depolarization of mitochondrial membrane after PAL application, which suggests that mitochondrial pathway is included in plasma action. It is in good agreement with the studies of lipid peroxidation of cell membranes, which are observed in plasma treatments too [45].

In the indirect plasma treatment of liquids, only the RONS created in PAL are responsible for the effects induced in the cells. Although the plasma short-lived species generated in PAL are already not present when the PAL are applied on cells, the secondary short-lived reactive species can be formed in the PAL from the long-lived RONS and their reactions with medium components. Our plasma source was used in the previous study to induce apoptosis in gastric cells (by using both direct treatment and PAM), cervix cancer cells and Ewig sarcoma cells (only direct treatment). A direct treatment of 30 s of the cells in a medium or the indirect treatment of 1 mL medium was enough to induce apoptosis. On the contrary, the apoptosis was not induced in human diploid fibroblasts (Alpha-1) after direct treatment for less than 1 min [28].

To explain the cancer versus normal cell selectivity effect, several possible models were developed. One of the first suggestions is that cancer cells have already a higher ROS level, so they can more easily reach the threshold when more exogenous RONS are added, and so lower RONS concentration is needed for the apoptosis induction [46]. This model is mostly not considered as valid. Another suggestion is that cancer cells have less cholesterol in their membranes, which increases the ROS influx into the cell [47]. Or, that cancer cells have more aquaporins in their membranes, so more H_2_O_2_ can enter the cell [48], but H_2_O_2_ must be kept in low concentration not to harm healthy cells. Although it is not confirmed, if aquaporins were the reason for the selectivity, it is certain that H_2_O_2_ plays an important role in the anticancer effect of PAM. Another recently published explanation of a selective plasma action towards the cancer cells is the action of extracellular singlet delta oxygen (^1^O_2_), which is either generated directly by the plasma discharge [49] or is formed in PAL by the reaction between the two main generated RONS—hydrogen peroxide and nitrites [9,12,50]. ^1^O_2_ formation proceeds through several steps involving peroxynitrite resulting from the reaction of H_2_O_2_ and NO_2_^−^ [26,51] with detailed reactions described in [9]. Because of this direct reaction between H_2_O_2_ and NO_2_^−^, we presumed that their stoichiometric ratio 1:1, in which they were formed in our plasma activated PBS, could be the best for the formation of peroxynitrites and consequently ^1^O_2_ [9]. Singlet oxygen can inhibit membrane-associated protective catalase on the cancer cells membranes. Subsequently it leads to the reactivation of HOCl signaling, which is RONS driven intercellular signaling, then H_2_O_2_ influx through aquaporins, activation of mitochondrial apoptotic pathway driven by caspase 3 and 9 [9], and a subsequent cell death through apoptosis [52]. With keeping low H_2_O_2_ concentration, this would lead to significant apoptotic induction in cancer cells without causing any harm to healthy cells. Moreover, the inactivation of membrane-associated catalase could lead to the generation of more secondary singlet oxygen, which would inactivate the membrane-associated catalases on the neighbouring cells and result in auto-amplificatory process spreading in the tumour [29,51].

Therefore, to verify the occurrence of the same mechanism in this work, PBS and medium enriched with externally added H_2_O_2_ and NO_2_^−^ was applied on the cells in approximately the same concentrations as were measured in PAPBS. However, these tests showed that H_2_O_2_ alone was capable of decreasing the viability of cancer cells (A375 and MiaPaCa-2) and, with these concentrations applied, the synergistic effect of reaction of H_2_O_2_ and NO_2_^−^ was not confirmed. Furthermore, externally added H_2_O_2_ was sufficient for apoptosis induction in cancer cells without any significant effect of NO_2_^−^ (data not shown). At the studied range of concentrations of H_2_O_2_ and NO_2_^−^, this agrees with the findings of Kurake et al. [10] and Sardella et al. [53], who indicated that NO_2_^−^ at higher concentrations (H_2_O_2_: NO_2_^−^ ratio 1:25 in [10] and 1:2 in [53]) also contributed to the selectivity of the antitumor effect of PAM. Although in the mentioned studies a higher concentration of NO_2_^−^ than H_2_O_2_ was required for the synergistic effect, in the model experiments [9] only a minimal concentration of nitrites (7.5 µM) was needed. Griseti et al. [34] confirmed synergistic effect with H_2_O_2_: NO_2_^−^ ratio 2:1, however at studied concentrations H_2_O_2_ alone was effective too and no comparison with non-cancer cells was made.

On the other hand, the important result of this study is that the low H_2_O_2_ concentration (60 µM) did not decrease the fibroblasts’ viability, neither in combination with NO_2_^−^. 30 µM NO_2_^−^ in 100% medium slightly decreased the viability of melanoma cells, while some viability increase was observed in 30 µM NO_2_^−^ in 50% PBS when applied on MiaPaCa-2. The cell stimulation effect of nitrites has already been published [54], but needs more investigation.

Our results presented here indicate the key role of low concentration H_2_O_2_ in the selective anticancer action of PAL. No apparent synergy with the same concentration of NO_2_^−^ was confirmed, which agrees with some authors who achieved comparable result only with H_2_O_2_ without NO_2_^−^ [12]. However, the PAL action is very complex, and it is still not yet fully clarified which RONS are dominantly responsible for the PAL anticancer action and which species may play additional roles. Therefore, we still cannot isolate specific RONS and conclude that their separate application without plasma treatment would lead to the same effects. As stated by Kurake et al., PAM exhibits a stronger cancer cell killing effect than only synergism appeared in medium spiked with H_2_O_2_ and NO_2_^−^, indicating that other latent mechanism should also be assessed [10].

In the current state of knowledge, cold plasma seems a very promising new option for cancer therapy [4] and other applications. It is a unique method to generate a mixture of RONS directly in an extracellular environment. The full understanding of the anticancer action of PAL and its selectivity certainly requires further research, including detailed insight into the RONS chemical interaction with cell growth media components.

## 4. Materials and Methods 

### 4.1. Cell Culture and Cultivation

Four adherent human cell lines were used to study the effect of plasma-activated liquids–melanoma cells A375 (Institute of Virology, Biomedical Research Center, Slovak Science Academy, Bratislava, Slovakia), pancreatic cancer cells MiaPaCa-2 (ATCC), glioblastoma cells LN229 (provided by Dr. Jinthe Van Loenhout and Prof. Evelien Smits from University of Antwerp, Antwerp, Belgium), and non-cancerous primary fibroblasts HDFa (provided by Dr. Jozef Hatok from Jesenius Faculty of Medicine, Comenius University, Martin, Slovakia). A375 and MiaPaCa-2 were cultivated in Dulbecco’s modified Eagle medium (DMEM, Sigma-Aldrich, St. Louis, MO, USA) supplemented with 10% fetal bovine serum (Sigma-Aldrich, St. Louis, MO, USA), LN229 in DMEM (Gibco, Invitrogen, Waltham, MA, USA) supplemented with 10% fetal bovine serum and 2 mM glutamine (Sigma-Aldrich, St. Louis, MO, USA) and HDFa cells in the fibroblasts growth medium (Cell Applications, San Diego, CA, USA). The cells were cultivated in 95% humidified atmosphere with 5% CO_2_ at 37 °C. 

The cells were inoculated in 96-well or 6-well plates according to the type of cell analysis, 24 h before the application of plasma activated liquids. 

Morphological changes of cells were observed, and pictures were taken by a microscope Zeiss Axio Verte.A1 (Zeiss, Jena, Germany) with a CCD camera Axiocam Icc 1 A1 (Zeiss, Jena, Germany) with Axio Vision software ((Zeiss, Jena, Germany, version 4.8, 2011).

### 4.2. Plasma Source and Plasma Liquid Activation

**Figure 16:**
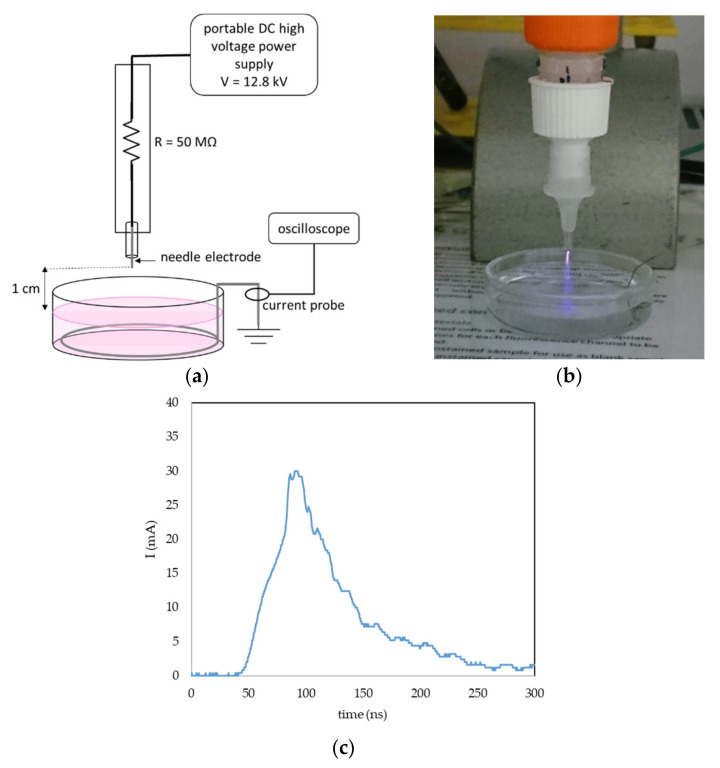
Streamer corona portable plasma pen in air and plasma activation of liquids. (**a**) General setup of the plasma pen in liquid activation; (**b**) photo of the PBS activation with streamer corona; and (**c**) example of one current pulse of the streamer corona discharge in the described geometry and voltage 12.8 kV.

A portable plasma pen was used for plasma treatment of liquids to create PAL (Figure 16a,b). A DC-positive streamer corona was generated in ambient atmospheric air between the tip of the needle electrode and the surface of the liquid. To keep the geometry of the system constant during the liquid activation, the liquid was placed in a glass bowl; the gap between the needle tip and surface of the liquid was 1 cm, and the volume of the liquid was 5 mL. The grounded stainless-steel wire was dipped in the liquid onto the bowl bottom and the current was measured with by a current probe (Pearson 2878, Pearson Electronics, Palo Alto, CA, USA) and a digital oscilloscope (Tektronix TDS2024, Tektronix, Beaverton, OR, USA). The applied DC voltage was set at 12.8 kV. The streamer corona in our setup is a self-pulsing discharge with a maximum amplitude of one pulse 20–30 mA (Figure 16c), an average frequency 10 kHz and a typical power 0.4 W. Because of the low temperature, power and current, it is possible to directly treat live cells or tissues, and even direct treatment of human skin is not painful when applied carefully.

Two different liquids were activated and applied to the cells—a phosphate-buffered saline (PBS, Dulbecco A, OXOID, Basingstoke, UK) and a cell growth medium (the same as used for the cell cultivation, supplemented as described in Part 4.1). Plasma-activated liquids (5 mL) were treated for 2.5, 5, 10 or 15 min. The pH, temperature and conductivity of the treated liquids did not change after plasma activation. 

To treat cells with plasma-activated PBS (PAPBS) or plasma-activated medium (PAM), various combinations of treatment times, added volumes or incubation times were used.

### 4.3. Chemical Analysis of RONS in PAPBS

Four different reactive species in PAPBS—H_2_O_2_, NO_2_^−^, NO_3_^−^ and ozone were measured by using spectrophotometric analysis. The absorption of samples was measured with a UV-VIS spectrophotometer UV-1800 Shimadzu (Shimadzu, Kyoto, Japan). 

H_2_O_2_ analysis was performed by the titanium oxysulfate assay. The concentration of hydrogen peroxide was calculated from the absorption (at 407 nm) of the yellow product formed in reaction between TiOSO_4_ and H_2_O_2_. To prevent H_2_O_2_ decomposition by its reaction with NO_2_^−^, sodium azide (NaN_3_, 60 mM) was added to the sample immediately after plasma treatment, as described in [37].

Nitrite (NO_2_^−^) concentration was evaluated with Griess reagents (Cayman Chemicals Nitrate/Nitrite Colorimetric Assay Kit #780001). NO_2_^−^ with Griess reagents forms a purple complex with the absorption maximum at 540 nm. To analyse nitrate (NO_3_^−^) concentration, nitrate reductase was added first to the sample to reduce NO_3_^−^ to NO_2_^−^, then analysed with Griess reagents as described before, and NO_3_^−^ concentration was calculated by the subtraction of previously measured NO_2_^−^ concentration.

Ozone dissolved in PAPBS was detected by using the indigo blue assay (Method 4500-O_3_) [55], which was mixed with the PAPBS immediately after plasma activation. The O_3_ decolorizes the indigo potassium trisulfonate dye and the concentration of ozone was calculated from the decrease of the absorbance at 600 nm. 

RONS in PAM have not been analysed due to its original pink colour and complex composition, which interacted with chemical reagents standardized to measure RONS in liquids.

### 4.4. Analysis of Cell Viability

The effect of PAL on cell viability was evaluated by using the colorimetric metabolic MTT (3-(4,5-dimethylthiazol-2-yl)-2,5-diphenyltetrazolium bromide) assay (Sigma-Aldrich, St. Louis, MO, USA). The assay is based on a change of the yellow MTT into the purple formazan in viable cells, which can be dissolved in dimethyl sulfoxide and spectrophotometrically analysed at 490 nm.

**Figure 17:**
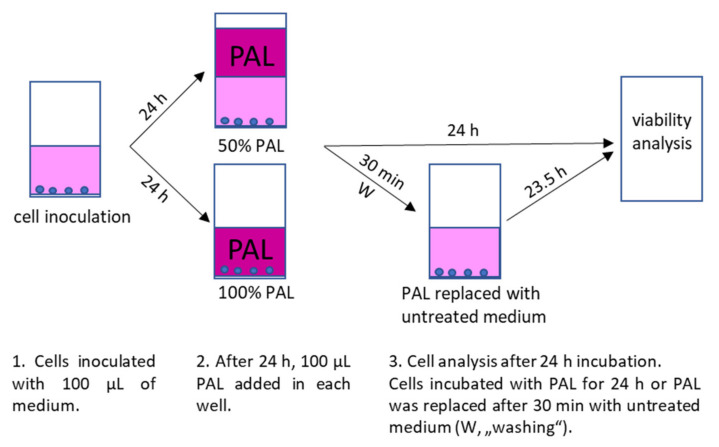
Experimental design of cell inoculation, plasma treatment after 24 h with 100% or 50% PAL and subsequent incubation with PAL for 24 h or only 30 min in PAL and 23.5 h in untreated medium (“washing”, W).

For inoculation, 10,000 cells (A375, LN229 and MiaPaCa-2) or 6000 cells (HDFa) in 100 µL medium per well of 96-well plates were used. The PAL were applied to the cells 24 h after their inoculation. Cell medium was either completely replaced with 100 µL PAL or PAL was added to the cells in a ratio 1:1 with already present medium, so the final concentration of PAPBS or PAM was 100% or 50%, respectively. The cells were incubated with PAPBS/PAM for additional 24 h or only 30 min and then replaced with an untreated medium for the rest of the time until the 24 h incubation (we called this treatment “washing”, marked as W in the graphs). The control cells were treated in the same way with untreated PBS and medium. The experimental design is shown in Figure 17.

The viability of the samples was normalized to the control cell sample incubated in the untreated medium.

### 4.5. Analysis of Apoptotic Cell Death with Annexin V

**Figure 18:**
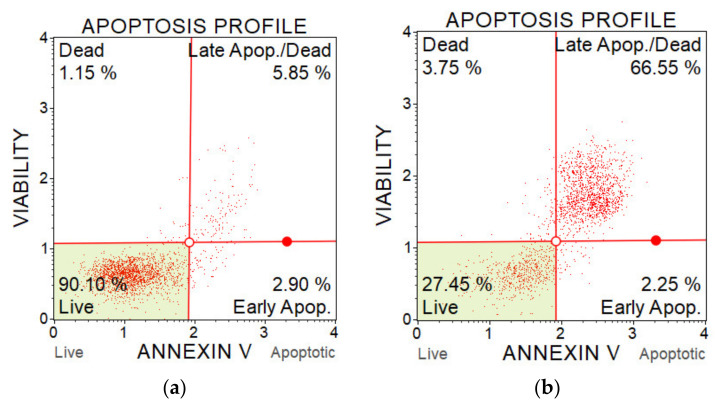
Flow cytometry plots from apoptosis analysis of A375 cells after PAM application. (**a**) Control sample (untreated medium); and (**b**) PAM activated for 5 min, 100% concentration.

The percentage of apoptotic cells was determined with a Muse Annexin V & Dead Cell Kit and a flow cytometer Muse Cell analyser (Luminex, Austin, TX, USA). The assay contains two fluorescent cell dyes—annexin V, which dyes phosphatidylserine located on the outer surface of cell membranes during apoptosis, and 7-AAD as an indicator of the cell membrane integrity. 7-AAD is excluded from live and early apoptotic cells but permeates later stage apoptotic and dead cells. The method enables to distinguish live cells, early apoptotic cells, late apoptotic cells and dead cells (which died in a non-apoptotic pathway). The better understand this method, the examples of results from two measurements are shown on Figure 18. 

Cells were inoculated in 6-well plates in amount of 250,000 (A375, LN229 and MiaPaCa-2) or 150,000 (HDFa) cells per well in 2.5 mL of liquid. The confluence and volume were kept proportional with the viability experiment. The cells were treated with PAL in the same way as described in Section 4.4, 24 h after inoculation, which means that the medium was either replaced with 2.5 mL PAL, or PAL was just added to the wells in a ratio 1:1 to the present medium (50% PAL). The cells were incubated with PAL either for 24 h or for 30 min, after which PAL was removed and cells were incubated in untreated medium until 24 h incubation (“washing”). The experimental design is the same as shown in Figure 17, only the volume of medium and PBS was 2.5 mL.

After an additional 24 h all the liquid from the well with already released and dead cells and cells from the bottom released by using trypsin were centrifuged, resuspended in 1 mL PBS, and prepared for the analysis according to the Muse kit protocol and analysed with Muse flow cytometer.

### 4.6. Analysis of Caspase 3/7 Activity

The apoptosis status was evaluated based on effector caspases activation—caspase-3 and caspase-7, which are considered as specific hallmarks of apoptosis. The caspase activity was measured with the Muse Caspase-3/7 Kit and the Muse Cell analyser. The kit contains 7-AAD dye to distinguish live and dead cells, and a Muse Caspase-3/7 reagent NucView to detect caspase activity. The Muse Caspase-3/7 reagent is cell membrane permeable and non-toxic to the cell. The Muse Caspase-3/7 reagent contains a DNA binding dye that is linked to a DEVD peptide substrate. When bound to DEVD the dye is unable to bind DNA. Cleavage by active Caspase-3/7 in the cell results in release of the dye, translocation to the nucleus and binding of the dye to DNA and high fluorescence. According to the fluorescent signal, the live cells, live with activated caspase-3/7 (apoptotic), dead with activated caspase-3/7 and dead cells (which died in non-apoptotic pathway) can be distinguished (as shown on Figure 18). The experimental design is the same as shown in Figure 17, only the volume of used PAL is 2.5 mL.

The cells were inoculated, treated and processed in the same way as described in Section 4.5, and dyed according to the Muse kit protocol and analysed with a Muse flow cytometer.

### 4.7. Analysis of Mitochondrial Potential Changes

The change in mitochondrial membrane potential—depolarisation, which is also considered as a hallmark of early apoptosis and cellular stress, was evaluated by using a MitoPotential Kit containing fluorescent MitPotential and 7-AAD dye. The MitoPotential Dye is a cationic, lipophilic dye used to detect changes in the mitochondrial membrane—high membrane potential drives accumulation of MitoPotential dye within the inner membrane of intact mitochondria, resulting in high fluorescence. Cells with depolarized mitochondria demonstrate a decrease in fluorescence and a downward shift. According to the fluorescent signal, the live cells, live cells with depolarized mitochondrial membrane, dead with depolarized mitochondrial membrane and dead without mitochondrial membrane depolarization can be distinguished (as shown in Figure 18).

The cells were inoculated, treated, processed in the same way as described in Section 4.5 and dyed according to the kit protocol.

### 4.8. The Effect of H_2_O_2_ and NO_2_^−^ on the Cells

To test the effect of two most discussed long-lived RONS, H_2_O_2_ (Sigma Aldrich, St. Louis, MO, USA) or NO_2_^−^ (added as NaNO_2_) or their combination were added into PBS or cell medium externally and applied to melanoma A375 cells, pancreatic cancer cells MiaPaCa-2 and human dermal fibroblasts HDFa.

The cells were inoculated and treated comparably as in the viability experiment described in Section 2.4. The cells were inoculated in 96-well plates in the amount of 10,000 cells per well (A375, MiaPaCa-2) or 6000 per well (HDFa) with 100 µL of the cell growth medium. After 24 h from inoculation, 100 µL of medium or PBS enriched with H_2_O_2_, NO_2_^−^ or their combination was added into the wells. Approximately the same average concentrations of H_2_O_2_ and NO_2_^−^ as those measured in PAPBS were used to compare with PAPBS effect (15 µM, 30 µM and 60 µM). We also added H_2_O_2_ and NO_2_^−^ at these concentrations to the medium; however, the resulting RONS concentration in PAM can be different. 

The cell viability was measured after an additional 24 h of incubation.

### 4.9. Statistical Analysis

Statistical analysis was performed in Microsoft Excel (Microsoft, Redmont, WA, USA, version 2106, 2016) and StatsDirect (StatsDirect, Birkenhead, UK, version 3, 2013). The normality of data was tested, and data are presented as the average and the standard deviation of the mean. At least three independent repeated experiments in each experimental condition were performed. The significance of plasma-activated liquids’ effect was tested with two-sided t-test and the result was considered as significant if *p* < 0.05. The significant results are marked with * in the graphs. 

## 5. Conclusions

Many recent studies showed that cold plasma can be successfully used against cancer cells, both in direct action as well as plasma-activated liquids. These liquids act through plasma-generated RONS. This study attempts to show that if the plasma treatment process is well tuned, PAL can induce selective apoptosis in cancer cells without damage to healthy cells.

A portable plasma pen operating with streamer corona in ambient air was applied to activate two types of liquids suitable for the application on human cells—the cell growth medium and PBS. We measured H_2_O_2_, NO_2_^−^ and NO_3_^−^ concentration in PAPBS. The ratio of generated H_2_O_2_ and NO_2_^−^, the species assumed to have the key roles in initiating apoptotic effects in cancer cells and its possible selectivity between cancer and normal cells, was approximately 1:1. This makes the main difference between PAL activated by our ambient air plasma pen and frequently used argon plasma jets.

Both PAM and PAPBS decreased the viability and induced apoptosis in three cancer cell lines—melanoma cells A375, pancreatic cells MiaPaCa-2 and glioblastoma cells LN229. The effect was confirmed for various plasma treatment times and PAL concentrations. We showed that even 30 min cell incubation in 50% diluted in medium PAL is sufficient to start processes leading to cancer cell apoptosis. Glioblastoma cells appeared to be the most sensitive to PAL. Besides apoptosis detection by standard annexin V staining, we also focused on more detailed insight on the apoptosis pathways by investigating the action of caspase 3/7 enzymes and mitochondrial membrane depolarization in melanoma cells. The effect of PAL on normal dermal fibroblasts was minimal, with only a little viability decrease for longer time treated PAPBS and with no detected apoptosis (or necrosis) induction. This indicates a generally desired selectivity of plasma and PAL dependent on correctly chosen plasma activation time and PAL concentration. However, we did not confirm the assumption of H_2_O_2_ and NO_2_^−^ synergy at their studied low concentrations and 1:1 ratio: even H_2_O_2_ alone in low concentration acted selectively and induced apoptosis only in cancer cells.

Cold air plasma, as a generator of specific RONS with anti-cancer capacity in liquids, including interstitial fluid, has a good potential for future in vivo studies and eventual clinical application in cancer treatments. More research is still needed to clarify the detailed mechanisms of its action.

## Data Availability

Data is contained within the article; more experimental data is available on request.

## Sample Availability

Samples of the PAM and PAPBS or compounds for RONS measurement are available from the authors.
